# Free Trade in Poisons

**Published:** 1847-06

**Authors:** 


*Free Trade in Poisons.*—A trial for manslaughter by poisoning with Godfrey's cordial took place at Guildford on Saturday last, and it came out on the cross-examination of one of the witnesses, that laudanum and Godfrey's cordial were sold at different shops in the town in common with *grocery, cheese, candles,* and *drapery!* One of the shopmen, in answer to a question put to him by the counsel for the defence, said very innocently that he did not know whether they kept prussic acid and arsenic, as well as laudanum, among their articles of grocery!

Not long since a superintendent of police went in disguise to a general shop in a country village and asked for two pennyworth of arsenic. It was immediately weighed in the scales in which coffee and other articles were weighed, and handed to him by the old woman who kept the shop. Upon asking her whether she was not in the habit of marking "poison" upon articles of this kind, her answer was: "Lor bless you, I can't write to begin with, and then it would frighten people if poison was written on it." This experiment was made in order to ascertain with what facility enough arsenic might be procured to destroy the lives of almost *one hundred persons:* and it was perfectly successful! It furnishes a clue to the cause of the abundant occupation given to coroners, magistrates, judges, barristers, and others, in detecting and punishing individuals for the perpetration of crimes which the Government takes not the slightest pains to prevent.—*Lond. Med. Gazette.*

